# The relationship and agreement between systemic and local breakpoints in locomotor and non-locomotor muscles during single-leg cycling

**DOI:** 10.3389/fphys.2025.1465344

**Published:** 2025-02-24

**Authors:** Markus Tilp, Nina Mosser, Gudrun Schappacher-Tilp, Annika Kruse, Philipp Birnbaumer, Gerhard Tschakert

**Affiliations:** ^1^ Institute of Human Movement Science, Sport and Health, University of Graz, Graz, Austria; ^2^ Institute of Electronic Engineering, FH JOANNEUM, University of Applied Science, Graz, Austria

**Keywords:** respiratory compensation point, near-infrared spectroscopy, electromyography, threshold, metabolic responses, physiological responses

## Abstract

**Introduction:**

There is a well-established relationship between the respiratory compensation point (RCP) and local muscular breakpoints determined from near-infrared spectroscopy (NIRS) and electromyography (EMG). However, these breakpoints have not yet been compared both in locomotor and non-locomotor muscles simultaneously in single-leg cycling exercise. Therefore, the aim of the study was to investigate the relationship and agreement between systemic and local breakpoints in locomotor and non-locomotor muscles.

**Method:**

Data from twelve physically-active participants (25.5 ± 3.9 years, 176.1 ± 11.6 cm, 71.2 ± 9.4 kg, 4 females) who completed a continuous single-leg step incremental cycling test (10 W min^-1^) with their right leg were included in the analysis. Ventilation and gas exchange were recorded to determine RCP. Surface EMG (sEMG) and NIRS signals were measured from both vasti lateralis muscles and breakpoints were determined from root mean Q square sEMG and deoxygenated hemo- and myoglobin signal m[HHb].

**Results:**

There was no significant difference in the power output at RCP (127.3 ± 21.8 W) and local muscular breakpoints both from the locomotor (m[HHb]: 119.7 ± 23.6 W, sEMG: 126.6 ± 26.0 W) and non-locomotor (m[HHb]: 117.5 ± 17.9 W, sEMG: 126.1 ± 28.4 W) muscles. Breakpoints also showed significant (p < 0.01) correlations (r = 0.67–0.90, ICC = 0.80–0.94) to each other with weaker correlations in the non-locomotor muscle (r = 0.66–0.86, ICC = 0.74–0.90). Despite the strong correlations, high individual variability and weak limits of agreement (up to −32.5–46.5 W) and substantial absolute differences (10.2–16.7 W) were observed which indicates that these breakpoints cannot be used interchangeably.

**Discussion:**

These findings offer further insights into the mechanistic relationship between local and systemic physiological response to exercise with increasing workload. We conclude that, despite strong correlations, local muscular breakpoints do not have to coincide with systemic boundaries of physiological domains.

## 1 Introduction

The determination of breakpoints (also referred as thresholds or turn points) to demarcate three intensity zones with distinctive metabolic properties (Binder et al., 2008; Hofmann and Tschakert, 2011) is relevant for exercise and sports practice. Several methodological approaches exist to determine these breakpoints. Most of these methods are based on systemic adaptions to increasing exercise intensity, e.g., based on blood lactate concentration, gas exchange variables, or heart rate (Meyer et al., 2005) while others are related to the local adaptions of the muscles. A common systemic breakpoint is the respiratory compensation point (RCP) which demarcates the transition from heavy to severe exercise intensity and is determined by the performance depending changes in the curse of oxygen uptake and carbon dioxide output in relation to ventilation (Beaver et al., 1986). The local breakpoints are based on muscular activity measured by electromyography (EMG) signals (e.g., root mean square EMG) or muscular oxygenation measured by near-infrared spectroscopy (NIRS signals) (Boone et al., 2016b). Typically, root mean square (RMS) values or mean power frequency from EMG (Ertl et al., 2016) and oxygenated (O_2_ [HHb]) and deoxygenated (m[HHb]) hemoglobin and myoglobin, total amount of tissue heme (totalHb), and tissue saturation (SmO_2_) values from NIRS (Perrey et al., 2024) are used to determine these breakpoints. It has been shown consistently that systemic and local breakpoints are observed at similar intensities during exercise testing. However, there is still an ongoing debate if there is a mechanistic relationship between local and systemic breakpoint concepts (Caen and Boone, 2023; Goulding et al., 2023).

Boone et al. (2016b) provided a theoretical framework that represents the relationship between muscular activity, metabolic processes, ventilation (VE), and (cerebral) blood flow which may explain the possible mechanistic link between different breakpoint concepts. Noticeably, apart from respiratory muscles that increase in activation due to the need for increased respiration (Contreras-Briceño et al., 2022), non-locomotor muscles are not included in this framework. However, several studies already investigated the behavior of non-locomotor muscles of the arm during lower limb exercises and reported increased blood flow (Tanaka et al., 2006) and decreased oxygenation in the inactive limb (Shiroishi et al., 2010; Özyener et al., 2012; Yogev et al., 2022; Sendra-Pérez et al., 2024a). This indicates that non-locomotor muscles are related to locomotor muscles through the systemic blood flow. Hence, local breakpoints in variables based on blood flow, e.g., based on NIRS signals should be observable in non-locomotor muscles. As a consequence, the framework between systemic and local breakpoints by Boone et al. (2016b) could be augmented by non-locomotor muscles. Indeed, Ogata et al. (2004), Yogev et al. (2022), and Sendra-Pérez et al. (2023) reported that breakpoints in NIRS signals of non-locomotor upper limb muscles during ramp leg exercises coincide with the respiratory compensation point (RCP) which is determined from systemic spirometry variables and demarcates the intensity for the transition from steady state to non-steady state exercise conditions. However, in contrast to breakpoints from NIRS signals, local breakpoints based on muscular activity (EMG) should not be observable in non-locomotor muscles as they are assumed to not increase activation during the increase of exercise intensity. Although such an observation would strengthen the framework by Boone et al. (2016b), this has not been tested yet.

Therefore, the aim of the study was to investigate the relationships and agreement between systemic and local breakpoints in locomotor and non-locomotor muscles. Our hypotheses were twofold: First, in the locomotor muscles we hypothesized a relationship and agreement between RCP determined from systemic variables and local muscle breakpoints based on muscular activity (sEMG, RMS) and oxygenation (NIRS, m[HHb]). Second, in non-locomotor muscles we hypothesized a relationship and agreement between RCP and the local breakpoint based on oxygenation (NIRS, m[HHb]) but not based on muscular activity (sEMG, RMS).

## 2 Methods

### 2.1 Participants

Boone et al. (2016a) reported a significant relationship (r = 0.91, p < 0.01) between the breakpoints of deoxygenated hemo- and myoglobin (m[HHb]_BP_) and integrated sEMG (EMG_BP_) signals. Based on these results, a minimum of eight participants was calculated (using G*Power software, Faul et al., 2007) to achieve a significant relationship between the breakpoints in our study. To account for technical problems and possible non-responders, 13 participants (4 females) were included in the study. Data from one participant had to be discarded due to a tattoo on the thigh, which affected the NIRS data. Therefore, 12 participants (4 females, 25.5 ± 3.9 years, 176.1 ± 11.6 cm, 71.2 ± 9.4 kg, skinfold thickness: 5.93 ± 0.8 mm, VO_2Peak_: 43.3 ± 4.1 mL.kg^−1^min^−1^, W_Peak_: 177.1 ± 29.9 W) were included in the final analyses. All participants were physically active but not specifically trained in cycling. Participants were eligible for the study if they were free of acute infections, injuries, chronic diseases, recent medication intake, or any restrictions that could have influenced the test. The study was approved by the local ethics committee (GZ. 39/132/63 ex 2022/23) and conducted in accordance with the Declaration of Helsinki.

### 2.2 Experimental design

Participants performed one maximal single-leg step incremental cycling test. They were instructed not to perform any strenuous exercise within 24 h before the test. On the test day, following the signing of the written informed consent, anthropometric measurements were recorded and the electromechanically braked cycle ergometer (Excalibur Sport, Lode, Groningen, Netherlands) was individually adjusted to the participant. The single-leg incremental cycling test was performed with the right leg in all participants. To enable a safe and easy execution of the test, the left pedal was demounted. The incremental protocol to exhaustion started with a 3-minute rest period followed by a 5-minute warm-up at 40 W. Subsequently, the load was stepwise increased by 10 W^.^ min^−1^and ended when the participants could not sustain the load any longer with a cadence of approximately 80 revolutions per minute. The test ended with a 3-minute cool-down period at 40 W followed by a 3-minute rest period. The cycle ergometer and the spirometry were electronically synchronized while sEMG and NIRS were manually synchronized with the rest of the devices by pushing the start button at the beginning of the measurements. Possible asynchronies should be clearly below 1 s and therefore irrelevant for the aim of the study.

### 2.3 Cardiopulmonary measurements

Expired air was continuously measured during the test with a breath-by-breath system (Metamax 3B, Cortex Biophysic Gmbh, Leipzig, Germany). The spirometer was calibrated according to the manufacture’s guidelines on every test day. Raw data was exported as excel CSV in 5 s intervals via Metasoft Studio software (Cortex Biophysic Gmbh, Leipzig, Germany). Heart Rate (HR) was measured using a chest strap (H10, Polar Electro, Kempele, Finnland) which was connected to the spirometer via Bluetooth signal.

### 2.4 Near-infrared spectroscopy

Relative changes in deoxygenated haemoglobin + myoglobin (m[HHb]) were measured at 10 Hz by a continuous wavelength portable NIRS device (PortaMon, Artinis Medical Systems, Elst, Netherlands) (Barstow, 2019). Positions for the NIRS sensors were at 1/3 the distance from the proximal pole of the patella to the greater trochanter (van der Zwaard et al., 2016). Prior to placing the NIRS sensors, adipose tissue thickness was measured with ultrasound (Esaote Mylab 60, Esaote SpA, Genova, Italy). The skin was then shaved and cleansed with alcohol. The NIRS sensors were wrapped in transparent foil to protect them from sweat and were attached with tape to the leg. Furthermore, a light-absorbing black cloth and elastic bandages were then wrapped around the thigh to shield the sensors from ambient light.

### 2.5 Electromyography

Muscle activity was assessed using sEMG (Ultium EMG System, Noraxon Inc., Scottsdale, AZ, United States) recording at a sampling rate of 2000 Hz (MR3 software version 3.18.64, Noraxon Inc., Scottsdale, AZ, United States). The sEMG electrodes were placed as close as possible proximally to the NIRS device, and ultrasound imaging was used to ensure that the electrodes were positioned on the VL muscle. Following established methodology (Hermens et al., 2000), the skin was prepared properly before electrode placement, including shaving, abrasion with sandpaper, and thorough cleansing with alcohol to optimize impedance conditions for accurate sEMG signal measurements. To minimize the risk of detachment during movement, the sEMG sensors were securely attached to the leg using double-sided adhesive tape under and strips over the sensors, while ensuring that the cables leading to the electrodes were not impeded.

### 2.6 Data analysis

#### 2.6.1 Systemic variables

Based on the actual standard three-phase two threshold model of energy supply (Skinner and Mclellan, 1980; Binder et al., 2008), two breakpoints were determined from an incremental protocol to exhaustion. Using respiratory parameters, the first ventilatory threshold/breakpoint (VT_1_), as well as the second ventilatory threshold/breakpoint (VT_2_), which is equal to the respiratory compensation point (RCP), were determined according to Beaver et al. (1986), Wasserman et al. (1994), and Binder et al. (2008). VT_1_ was defined as the first increase of VE accompanied by an increase in $\dot{V}$ E/ $\dot{V}$ O_2_ without an increase in $\dot{V}$ E/ $\dot{V}$ CO_2_. VT_2_ respectively RCP, which was used for further analyses, was determined by an increase in both the respiratory equivalent for oxygen ( $\dot{V}$ E/ $\dot{V}$ O_2_) and for carbon dioxide ( $\dot{V}$ E/ $\dot{V}$ CO_2_) accompanied by the second sharp increase in $\dot{V}$ E detected by means of multi-linear regression analysis using Vienna CPX-Tool (https://www.univie.ac.at/vcpx/), a commercially available software. The region of interest for determination of RCP was set between the first threshold/breakpoint (approximately 40% of peak power output) and peak power output (W_peak_) which denotes the highest power output pedaled for at least 30 s in the incremental test. The highest VO_2_ value averaged over a 30 s period at W_peak_ represented $\dot{V}$ O_2peak_. Since the specific one-leg exercise modality did not allow a maximum systemic exhaustion, and since the determination of VO_2max_ was not targeted in this investigation, the termsVO_2peak and_ W_peak_ were used.

#### 2.6.2 Local muscle variables

Sample rate and the noise of the m[HHb] NIRS signal was reduced by a factor of 50 using a lowpass Chebyshev Type I infinite impulse response filter of order 8. Root mean square (RMS) of the raw sEMG signals were calculated using a sliding window of 1,000 points corresponding to a window duration of 9.5 s. Subsequently, the sample rate and the noise of the sEMG signals was reduced by a factor of 1,000 using a lowpass Chebyshev Type I infinite impulse response filter of order 8. Then, a two-line regression (Osawa et al., 2011; Boone et al., 2016a) was employed for both NIRS and sEMG data to determine a possible breakpoint (m[HHb]_BP,_ EMG_BP_) with the lowest overall root mean square error. As artefacts were observed at the beginning and the end of the measurements, the first 3 minutes when workload started to increase and the last 2 minutes of the data before maximal workload was reached were not included. Furthermore, the region of interest to determine the breakpoint was limited to 40%–90% of peak power output. A 50-fold resampling strategy to ensure the robustness and objectivity of the detected breakpoints was used. Specifically, 80%of the data set was randomly sampled to estimate the breaking point in each fold. The mean value of the breaking points from these folds provides a robust and objective estimate of the physiological breakpoints.

### 2.7 Statistical analysis

Means, standard deviations, and 95% confidence intervals were calculated for all variables. A Shapiro-Wilk test was used to test for normal distribution and a Mauchly-test was used to test for sphericity. Power output data was normally distributed (p = 0.16–0.95 for different variables) but sphericity was violated (p < 0.01), therefore, a Greenhouse-Geissler correction was applied for the repeated measures analysis of variance to compare all the means (RCP, EMG_BP_ and m[HHb]_BP_) of the locomotor (right vastus lateralis) and non-locomotor (left vastus lateralis) muscles. Pearson correlation coefficients were computed to evaluate the linear relationship between the power output values at which the breakpoints occurred across the variables. According to Cohen (1988), the magnitude of correlations was assessed as small, medium, and strong for r = 0.10, r = 0.30, and r = 0.50, respectively. Furthermore, intraclass correlation coefficients (ICC(3.1)) were computed between breakpoints to assess the agreement between breakpoints. Bland-Altman analysis (Bland and Altman, 1986), mean absolute difference, and regression intercepts were used to assess agreement in power output between the breakpoints. All tests were performed with SPSS 29 and the level of significance was set to 0.05.

## 3 Results

### 3.1 Locomotor muscle

**FIGURE 1:**
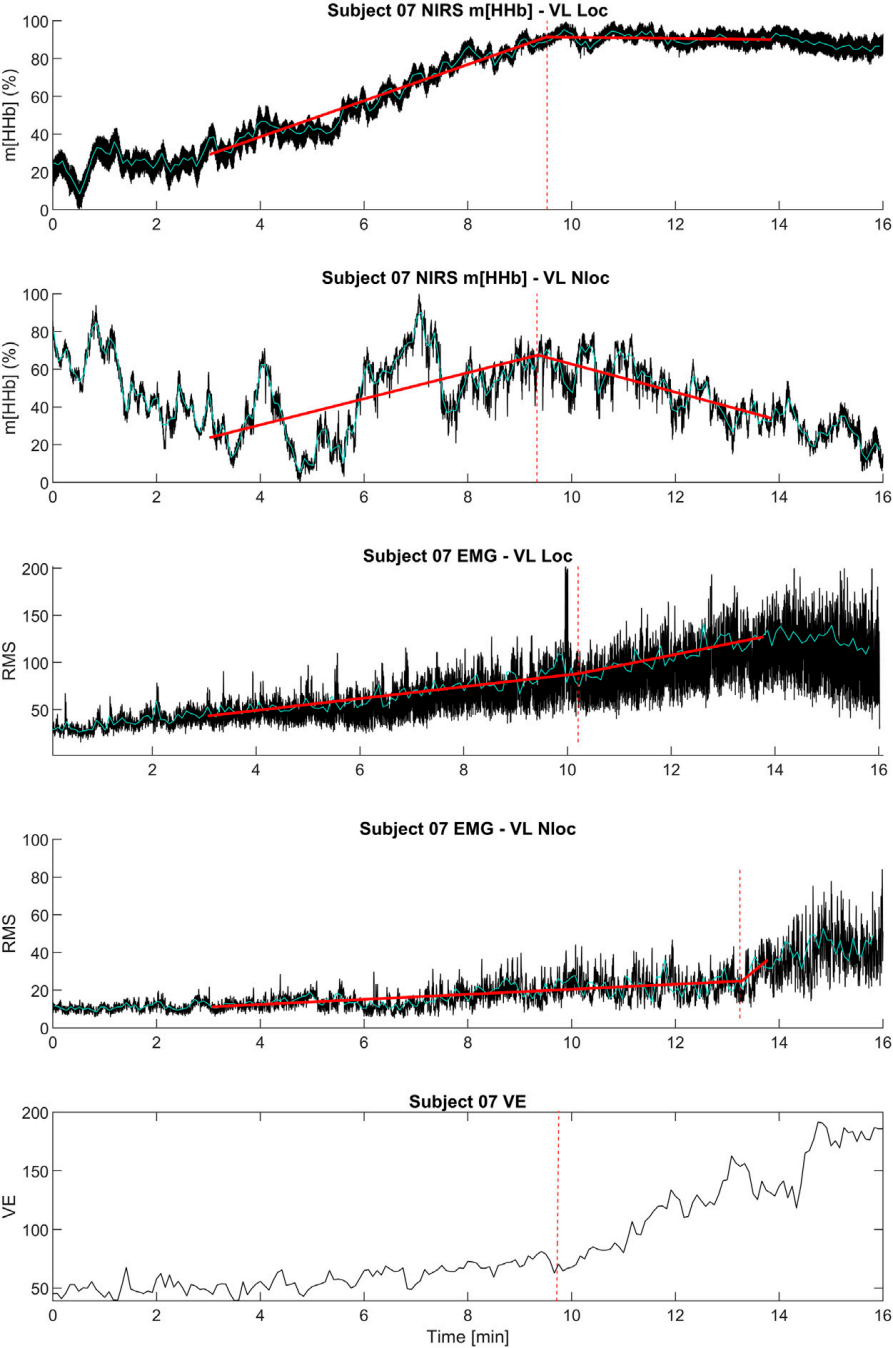
Exemplary data set of an individual subject including the kinetics of ventilation ( $\dot{V}$ E), and the locomotor (Loc) and non-locomotor (Nloc) vastus lateralis (VL) NIRS (HHb) and EMG (RMS) signals during the step-incremental single-leg cycling. Black lines show original data, cyan lines show filtered data, red lines show linear regressions. Vertical dashed red lines show breakpoints. Please note that HHb kinetics of the non-locomotor vastus lateralis does not represent a typical behavior as m[HHb] increased or decreased close to RCP in the different participants.

The m[HHb] signal in the working muscle (locomotor vastus lateralis) showed a consistent pattern that increased from the start and attenuated (or decreased) at about 75% of peak power output (W_Peak_). The sEMG (RMS) signal of the working muscle initially increased with increasing work rate, demonstrating a distinct change in slope close to the RCP. However, this change in slope was not always an increase but in some cases a decrease in slope. Figure 1 shows a representative data set from an individual.

Table 1 shows the occurrence of the different breakpoints in W and as percentage of peak power output reached during the single-leg exercise. Furthermore, it shows the absolute values of VO_2_ and heart rate at breakpoints.

**TABLE 1:** Absolute and relative power values and absolute VO_2_ and HR values at the respiratory compensation point as well as m[HHb]_BP_ and EMG_BP_ of the locomotor and non-locomotor muscles.

	Power at breakpoints (W)		% of W_peak_ (%)		VO_2_ at breakpoints (L/min)	HR at breakpoints (bpm)
	Mean ± SD (CI, 95%)	Range	Mean ± SD (CI, 95%)	Range	Mean ± SD (CI, 95%)	Mean ± SD (CI, 95%)
RCP	127.3 ± 21.8 (113.5, 141.2)	94.6–167.5	71.9 ± 1.9 (64.1, 79.8)	68.1–74.5	2.24 ± 0.4 (1.99, 2.49)	161.5 ± 15 (151.8, 171.2)
m[HHb] (VL, Loc)	119.7 ± 23.6 (104.6, 134.7)	91.4–154.9	67.6 ± 6.7 (59.1, 76.1)	58.1–80.3	2.11 ± 0.4 (1.87, 2.36)	158.8 ± 14 (149.9, 167.8)
m[HHb] (VL, Nloc)	117.5 ± 17.9 (106.1, 128.8)	93.2–156.4	66.9 ± 7.2 (60.4, 73.3)	55.0–78.2	2.16 ± 0.3 (1.97, 2.36)	158.1 ± 12 (150.6, 165.5)
EMG (VL, Loc)	126.6 ± 26.0 (110.2, 143.1)	88.1–171.1	71.3 ± 5.6 (62.1, 80.6)	59.8–81.5	2.22 ± 0.4 (1.95, 2.49)	162.0 ± 15 (152.3, 171.8)
EMG (VL, Nloc)	126.1 ± 28.4 (108.1, 144.2)	88.6–179.5	70.9 ± 7.4 (60.8, 81.1)	58.0–81.5	2.25 ± 0.5 (1.95, 2.56)	162.2 ± 13 (154.0, 170.5)

Data are presented as mean ± SD, w_peak_ peak power; HR, heart rate; SD, standard deviation; RCP, respiratory compensation point; Loc, locomotor muscle, Nloc non-locomotor muscle; VL, vastus lateralis.

**FIGURE 2:**
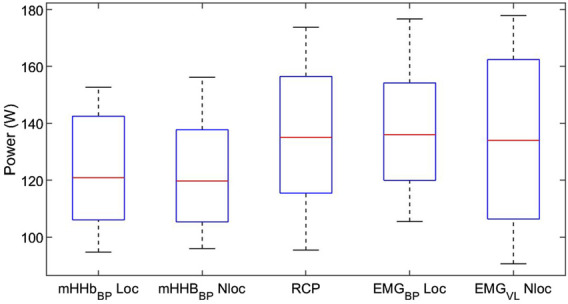
Mechanical power at breakpoints of different variables. Note that there was no significant difference between the breakpoints (boxes show the range of the central 50% of data, red lines display the medians, whiskers display minimum and maximum values of data).

**FIGURE 3:**
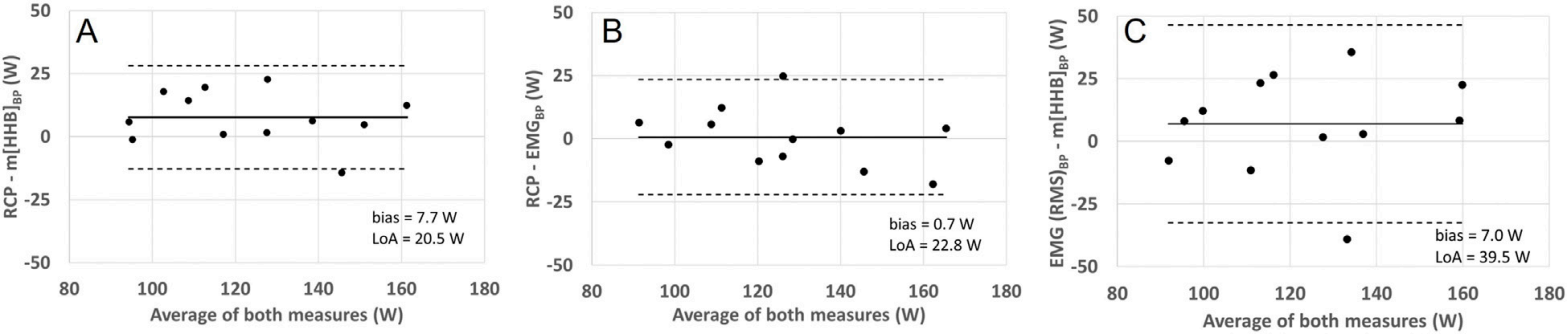
Bland-Altman plots displaying agreement between power (W) corresponding with RCP and VL loc m[HHb] **(A)**, RCP and VL Loc EMG BP **(B)**, and VL Loc EMG BP and VL Loc m[HHb] **(C)**. The horizontal solid line represents the mean difference and the horizontal dashed line the 95% limits of agreement.

Repeated measures analysis of variances with Greenhouse-Geiser correction indicated that there was no significant difference between power output values at RCP and breakpoints of the m[HHb] and sEMG responses of the locomotor and non-locomotor vastus lateralis, F (3.0,33.4) = 1.53, p = 0.23, η^2^p = 0.122 (see Figure 2). Figure 3 presents Bland-Altman plots displaying the agreement between power output at RCP, m[HHb]_BP_, and EMG_BP_ of the locomotor vastus lateralis. The mean average difference between RCP and m[HHb]_BP_ was 7.7 W (limits of agreement (LoA): lower = −12.8 W, higher = 28.1 W) with a mean absolute difference of 10.2 ± 7.7 W. The mean average difference between RCP and EMG_BP_ was 0.67 W (LoA: lower = −22.1 W, higher = 23.4 W) with a mean absolute difference of 8.8 ± 7.1 W. The mean average difference between m[HHb]_BP_ and EMG_BP_ was 7.0 W (LoA: lower = −32.5 W, higher = 46.5 W) with a mean absolute difference of 16.7 ± 12.5 W.

**FIGURE 4:**
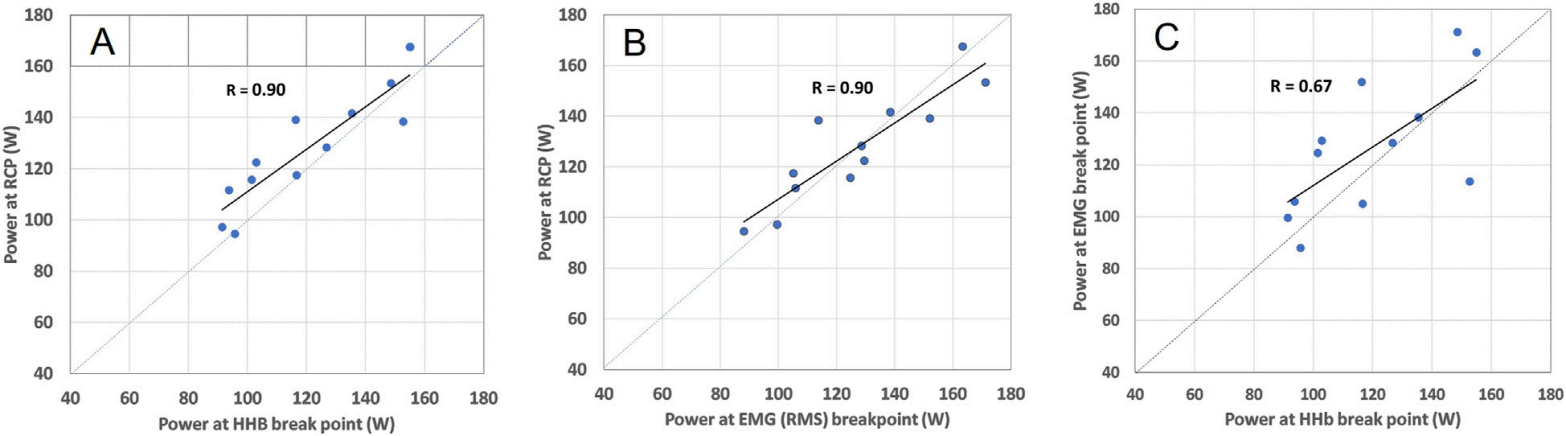
Relation between m[HHb]_BP_ and RCP **(A)**, EMG_BP_ and RCP **(B)**, and m[HHb]_BP_ and EMG_BP_ **(C)** in the locomotor VL including respective Pearson correlation coefficients. The dashed lines represent the line of identity.

Power output values of RCP and all breakpoints determined from the locomotor vastus lateralis correlated significantly with each other (all p < 0.01). The correlation coefficients were r = 0.67 (0.16, 0.90) (m[HHb]_BP_ VL loc vs. EMG_BP_ VL loc), r = 0.90 (0.67, 0.97) (m[HHb]_BP_ VL Loc vs. RCP), and r = 0.90 (0.66, 0.97) (RCP vs. EMG_BP_ VL Loc). Intraclass correlation coefficients (ICC(3.1)) were ICC = 0.80 (0.34, 0.94) (m[HHb]_BP_ VL loc vs. EMG_BP_ VL loc), ICC = 0.92 (0.63, 0.98) (m[HHb]_BP_ VL Loc vs. RCP), and ICC = 0.94 (0.80, 0.98) (RCP vs. EMG_BP_ VL Loc). The relationships between the RCP as well as m[HHb]_BP_ and EMG_BP_ from the locomotor VL are shown in Figure 4. Bias assessed as regression intercepts were not significant (Table 2).

**TABLE 2:** Pearson correlation coefficients, intraclass correlation coefficients, and power differences between breakpoints. Means (CI, 95%)/± SD.

	Correlation coefficient	ICC (3.1)	Bias/regression intercept [W]	LoA [W]	MAD [W]
RCP vs. m[HHb]_BP_	r = 0.90 (0.67, 0.97) (p < 0.01)	ICC = 0.92 (0.63, 0.98) (p < 0.01)	28.3 ± 15.7 (p = 0.10)	7.7 (−12.8, 28.1)	10.2 ± 7.7
RCP vs EMG_BP_	r = 0.90 (0.66, 0.97) (p < 0.01)	ICC = 0.94 (0.80, 0.98) (p < 0.01)	32.0 ± 15.2 (p = 0.06)	0.67 (−22.1, 23.4)	8.8 ± 7.1
m[HHb]_BP_ vs EMG_BP_	r = 0.67 (0.16, 0.90) (p < 0.05)	ICC = 0.80 (0.34, 0.94) (p < 0.01)	38.1 ± 31.2 (p = 0.25)	7.0 (−32.5, 46.5)	16.7 ± 12.5

LoA, Level of Agreement (Bland Altman), ICC, intraclass correlation coefficient; MAD mean absolute difference.

Similar to power output values, repeated measures ANOVAs revealed that there was no significant difference between RCP, m[HHb] _BP_, and EMG_BP_ in VO_2_ (F (4,44) = 0.53, p = 0.71, η^2^p = 0.046) and heart rate (F (4,44) = 1.01, p = 0.414, η^2^p = 0.084). For detailed results on heart rate and VO_2_ data, please see supplement.

### 3.2 Non-locomotor muscle

In contrastto the locomotor muscle, the non-locomotor muscle did not show such a consistent pattern in the m[HHb] signal. Here, the m[HHb] showed greater fluctuation with both increases or decreases after the RCP. The sEMG (RMS) signal of the non-locomotor muscle showed much lower absolute values and, similar to the non-locomotor m[HHb] signal, an inconsistent pattern (see Figure 1).

The m[HHb]_BP_ and EMG_BP_ from the non-locomotor muscle also correlated significantly, but not as strong, with RCP (r = 0.66 (0.13, 0.89)/ICC = 0.74 (0.16, 0.92), r = 0.77 (0.36, 0.93)/ICC = 0.90 (0.63, 0.97), respectively). Furthermore, there was a significant correlation between m[HHb]_BP_ and EMG_BP_ of the non-locomotor muscle with r = 0.86 (0.55, 0.96)/ICC = 0.79 (0.31–0.94).

## 4 Discussion

Several studies compared a systemic breakpoint with local muscle breakpoints derived from both NIRS and sEMG signals (Osawa et al., 2011; Racinais et al., 2014; Boone et al., 2016a; Iannetta et al., 2017; Inglis et al., 2017; Goulding et al., 2021; Caen et al., 2022). This study expands on previous research by including local muscle breakpoints from both the locomotor muscle and the contralateral non-locomotor muscle. As hypothesized, strong correlations between systemic breakpoints (RCP) and local muscular EMG_BP_ and m[HHb]_BP_ in the locomotor muscle (r = 0.90, ICC = 0.92–0.94) were observed with no significant differences between the power output at breakpoints. Although breakpoints were not significant different, substantial mean absolute difference values between systemic and local breakpoints (8.8–10.2 W) were observed. In the contralateral non-locomotor muscle, both m[HHb]_BP_ and, in contrast to our expectations, also EMG_BP_ correlated significantly with systemic RCP. However, correlation between breakpoints was smaller in the non-locomotor muscles (r = 0.66–0.77, ICC = 0.74–0.90) than in the active, main locomotor muscle. Also, in non-locomotor muscle no significant differences were observed between breakpoints.

### 4.1 Locomotor muscle

During an incremental exercise, an increasing number of motor units (from Type I → Type IIa → Type IIx) are recruited in the locomotor muscle (Henneman, 1957). This can be observed in the sEMG signal by an increased amplitude which further increases when type IIx fibers are recruited (EMG_BP_). Within the muscle, O_2_ extraction increases, leading to a decrease in m [O_2_Hb] and an increase in m[HHb]. With progressive recruitment of Type II fibers, it appears that O_2_ extraction reaches its limits and a plateau occurs in m[HHb], which allows to detect a m[HHb]_BP_ (Boone et al., 2016b). Furthermore, the progressive recruitment of motor units induces metabolic acidosis including H+ and lactate production in the muscle which will be transported to the blood system. The decrease of pH level from H+ will increase ventilation to maintain the acid-base balance which can be observed by the second inflection of the linear increase in ventilation at RCP (Wasserman et al., 1994).

When comparing systemic RCP and local muscle breakpoints for EMG_BP_ or m[HHb]_BP_, our results in the locomotor muscle (VL right leg) align with prior studies on cycling that demonstrated a strong correlation (Racinais et al., 2014; Boone et al., 2016a; Iannetta et al., 2017; Goulding et al., 2021). The correlation magnitudes in our study were similar or slightly lower to earlier findings. While we found a correlation between RCP and EMG_BP_ of r = 0.90, others reported correlations as high as 0.97 (Iannetta et al., 2017). The correlation between RCP and NIRS breakpoints with r = 0.90 was higher than r = 0.57 reported by (Racinais et al., 2014) but similar to other reported r-values ranging between 0.90–0.96 (Boone et al., 2016a; Goulding et al., 2021). As reported in the review by Sendra-Pérez et al. (2023), high relationships are also observed in other muscles (e.g., gastrocnemius) and different types of movements (e.g., running or rowing) with a combined intraclass correlation coefficient of 0.80 between NIRS breakpoints (based on muscle oxygen saturation SmO_2_) and the 2^nd^ ventilatory breakpoint.

In the present study, no significant differences between RCP and the local breakpoints were observed. However, differences in the occurrence and sequence of breakpoints across studies remain heterogeneous in the literature. For instance, some researchers reported that EMG breakpoints were detected earlier than RCP and NIRS breakpoints, with no significant differences among the latter (Boone et al., 2016a; Goulding et al., 2021). Conversely, other studies detected EMG breakpoints significantly later than both NIRS breakpoints and RCP (Osawa et al., 2011; Racinais et al., 2014). Moreover, in accordance to the present study, Iannetta et al. (2017) reported no difference between the three types of breakpoints.

This variability in breakpoints can partly be attributed to differences in the experimental protocol, such as with single-leg versus classic cycle ergometer tests, and data analysis methods. For example, some studies employed an individual Mean Response Time (MRT), adjusting for the delay in local metabolic responses reaching pulmonary circulation, i.e., the onset of $\dot{V}O_{2}$ after the onset of the incremental test (Fontana et al., 2015; Caen et al., 2018). This time duration is often used to align NIRS and EMG data with $\dot{V}O_{2}$ data inducing breakpoint upward shifts of 41–44 s (Boone et al., 2016a; Caen et al., 2022) towards RCP. Although we did not observe a significant difference in our data, m[HHb]_BP_ occurred 7.7 W earlier than RCP, which corresponds to approx. 46 s, similar to previously reported MRT values.

Furthermore, the methods for determining breakpoints varied, from visual inspection of RCP (Racinais et al., 2014; Boone et al., 2016a; Iannetta et al., 2017) to semi-automated methods utilized in this study. In addition, we modelled sEMG data kinetics with a two-line regression in accordance to Osawa et al. (2011) and Boone et al. (2016a) but in contrast to others (Hug et al., 2003; Iannetta et al., 2017) who account for a first and second EMG breakpoint. To summarize, while there is a strong underlying physiological mechanism shared across studies, the results are not interchangeable due to methodological differences and variations in experimental protocols.

### 4.2 Non-locomotor muscle

The presence of physiological breakpoints in non-locomotor muscles offers intriguing insights into the mechanistic relationship between local and systemic physiological response to exercise with increasing workload. However, although meaningful m[HHb]_BP_ and EMG_BP_ could be detected in all participants, the kinetics of m[HHb] and EMG was not as consistent in the non-locomotor compared to the locomotor muscles. This was probably due to different activation patterns in the different participants of which some showed clear muscle activity in the non-locomotor muscle, e.g., to maintain stability on the ergometer. Therefore, m[HHb] sometimes increased (in 7 out of 12 participants), attenuated (1), or decreased (4) close to the RCP. This inconsistency was similar in the sEMG kinetics, however, not directly related to m[HHb].

The presence of a NIRS breakpoint in a non-locomotor muscle can be explained by several mechanisms which may lead to opposing effects in the m[HHb] signal: 1. A systemic increase of blood flow in the non-locomotor (Tanaka et al., 2006) might increase O_2_ delivery and therefore decrease m[HHb]. 2. The re-distribution of blood flow during exercise favoring working muscles, respiratory muscles, and the brain would lead to a decrease in O_2_ delivery and therefore increase m[HHb] (Ogata et al., 2004). 3. The changes in HHb concentration from the locomotor (and respiratory) muscles are transported via the systemic blood flow to the non-locomotor muscle (Özyener et al., 2012; Yogev et al., 2022; Sendra-Pérez et al., 2024a). Hence, the m[HHb] might show a similar behavior as in the locomotor muscle. Close to the RCP this could lead to an attenuation or even a decrease of m[HHb]. 4. NIRS breakpoints could be attributed to the minor yet increasing muscle activity needed to stabilize the body’s position through co-contraction. For instance, during single-leg cycling, the contralateral leg’s muscles might not be directly involved in the pedaling action but play a crucial role in maintaining stability and distributing load, which could lead to changes in activity and would also lead to an increase m[HHb]. This was already hypothesized by several authors (Özyener et al., 2012; Yogev et al., 2022; Sendra-Pérez et al., 2024a) but muscle activity was not tested because of the lack of EMG measurements in these studies. In the present study, we could clearly observe co-contraction in the sEMG-data of the non-locomotor muscles (see also Figure 1). Although absolute sEMG values were much lower compared to those of the locomotor muscle, muscular activation that increased with increasing workload was present. This result contrasted findings from Tanaka et al. (2006) who did not observe any sEMG activity on the non-working muscle and was therefore not anticipated in our experiment. The minor but consistent activation of the non-locomotor muscles in the present study also led to the non-expected occurrence of EMG breakpoints which were significantly related and not different to RCP or other breakpoints. Hence, our hypothesis that non-locomotor muscles will not show EMG breakpoints must be rejected due to the reasons explained. Therefore, our results cannot augment the physiological framework by Boone et al. (2016a) by non-locomotor muscles yet. However, for future studies, we recommend to better control the activity of non-locomotor muscles.

### 4.3 Practical applications and physiological mechanism

The use of local breakpoints derived by NIRS (Murias et al., 2013), sEMG (Hug et al., 2003), or both (Iannetta et al., 2017) to estimate systemic breakpoints as a marker between heavy and severe exercise intensity has been studied extensively. While a general relationship with strong correlations has been shown consistently, a high degree of individual variability with large limits of agreement between the breakpoints suggest that systemic and local breakpoints should not be used interchangeably. This is supported by findings that a) training induced changes in systemic breakpoints (RCP) were not related to changes in NIRS breakpoints (Caen et al., 2018; Caen et al., 2022) and b) NIRS breakpoints from the same muscle differed between exercises in different body positions (Goulding et al., 2021). Our results support these conclusions as we also observed large limits of agreement between the breakpoints. Such results led to an ongoing discussion about a possible mechanistic link between local and systemic breakpoints (Caen and Boone, 2023; Goulding et al., 2023). Based on previous and our findings in the present study, we assume that the mechanistic link between the different breakpoints is the relationship between single muscles and the overall systemic cardio-respiratory and metabolic responses. With increased workload, physiological events like the sequential recruitment of muscle fibers combined with maximal O_2_ extraction occur in the working muscle and its effects are then transferred to the (cardio-respiratory and metabolic) system and non-working muscles. A comparison between local and systemic breakpoints is insofar difficult as many different muscles are involved in complex (whole body) movements like running or cycling which cumulatively create a systemic response (Yogev et al., 2022). The contribution of a single muscle to the systemic response is dependent on the activation and the size of a muscle and, therefore, dependent on intermuscular coordination. Although the contribution of bigger muscles to systemic responses should be generally greater than from smaller muscles, even behavior of smaller muscles during single joint movements (Spendier et al., 2020; Tilp et al., 2022) can be observed in systemic measures. During a movement with several (bigger and smaller) muscles involved, these muscles can be activated differently to share the work load. This will lead to very distinct activation patterns which are difficult to anticipate. Some muscles might increase their activation continuously until exhaustion while others might attenuate or decrease their activation at a certain point because other muscles take over their share of load. In the present study, we could observe all different types of sEMG-profiles (increasing, decreasing, attenuating) in the working muscle (VL) close to the power output at RCP.

The described relationship between the behavior of single muscles and systemic response can explain several unclear observations from the literature. Firstly, although the systemic conditions are mostly driven by the metabolic processes of the main working muscles it is not always possible to draw conclusions to single working muscles from systemic measures, especially in complex exercise where several muscles are involved. Systemic measures represent the cumulative effect of several muscles engaged in a specific movement. When these muscles with different sizes display individual local breakpoints at different workloads during a task with increasing effort, their cumulative systemic response may not accurately mirror their individual behavior. Sendra-Pérez et al. (2024a) recently showed nicely the heterogeneity in individual breakpoints from SmO_2_ of different muscles (see their Figure 2), although their means from 26 athletes were not significantly different. Conversely, if these muscles display their local breakpoints at the same workload, this breakpoints may coincide with the systemic breakpoint, possibly delayed by the time of the systemic response (Fontana et al., 2015). Hence, local breakpoints from individual muscles may (Snyder and Parmenter, 2009; Sendra-Pérez et al., 2023) or may not (Possamai et al., 2024; Arnet et al., 2025) coincide with systemic boundaries of physiological domains, depending on type of sport and intermuscular coordination. Secondly, training-induced changes in systemic variables (e.g., increase in RCP) must not necessarily be related to changes in local responses of a specific muscle tested as observed, e.g., by Caen et al. (2022). The training-induced improvements are likely related to the structural and functional improvements of several muscles and also related to improved intermuscular coordination. Thirdly, local muscle breakpoints from specific muscles must not necessarily appear at the same instant when determined from similar exercises in different positions. Goulding et al. (2021) observed different NIRS breakpoints during cycling in a sitting or supine position and they concluded due to their observation that RCP and NIRS breakpoints do not represent the same underlying physiological phenomenon. However, different body positions lead to different muscle lengths and contraction velocities and therefore to favorable or unfavorable contractile conditions for different muscles, therefore to different neuromuscular activation (Hug and Dorel, 2009), probably also depending on the individual anthropometry. Hence, it is not surprising that local breakpoints from a specific muscle show different kinetics in different body positions.

Although local muscular breakpoints cannot be used interchangeably with systemic breakpoints due to the reasons explained above, determining these breakpoints can be of great value. During movements where several muscles are involved, determining local breakpoints from several muscles could help in understanding which muscles are stressed earlier than others and therefore represent a bottleneck for improved performance. Specific training of these muscles could then improve overall performance. Exemplary types of sport would, e.g., be rowing or climbing, where both leg and arm muscles are responsible for overall performance. Furthermore, the effect of athletes’ position on different muscles during exercise could be tested to determine efficient movement conditions for specific muscles.

### 4.4 Limitations

Although an *a priori* power analysis has been performed, the sample with 12 participants including males and females with heterogenous performance levels limits the generalization of results. Furthermore, the results of the breakpoint determination depend crucially on the applied model and model constraints. However, the applied models and constraints are common in the literature. Contrary to our expectations, the non-locomotor muscle exhibited inconsistent activity in stabilizing the movement, thereby impacting the results. Unilateral cycling is very specific exercise which is technically difficult and the test is very much limited by the ability of the participants to pull the pedal up (hip flexor). For similar future experiments we recommend a counterweighted single-leg exercise (Iannetta et al., 2019) or a more isolated exercise as applied by Spendier et al. (2020). In general, the measurement of a single leg could have affected our results as wide limits of agreement in SmO_2_ values have been reported between the dominant and non-dominant leg during incremental cycling (Skotzke et al., 2024; Sendra-Pérez et al., 2024b). However, Iannetta et al. (2019) reported no difference in [HHb] breakpoints between the dominant and non-dominant leg during single-leg or counterweighted single-leg exercise.

### 4.5 Conclusion

In the locomotor muscles, the study revealed strong correlations between systemic breakpoints, particularly the respiratory compensation point (RCP), and local muscle breakpoints derived from both muscular activity (EMG) and oxygenation (NIRS signals). However, high individual variability and substantial absolute differences were observed which indicates that these breakpoints cannot be used interchangeably. Non-locomotor muscles exhibited varying behaviors in the signals, with m[HHb] and sEMG showing inconsistent patterns. However, meaningful m[HHb]BP and EMGBP were detected in non-locomotor muscles and correlated significantly with systemic RCP. These findings emphasize the complexity of the interplay between systemic and local physiological responses during exercise, highlighting the intricate nature of these relationships based on individual muscle coordination.

## Data Availability

The raw data supporting the conclusions of this article will be made available by the authors, without undue reservation.
