# Supplementary material for: The relationship and agreement between systemic and local breakpoints in locomotor and non-locomotor muscles during single-leg cycling
Source: Front Physiol. 2025 Feb 24;16:1465344. doi: 10.3389/fphys.2025.1465344 (PMC11891192; doi:10.3389/fphys.2025.1465344)
Supplement: Supplementary file 1 [file Presentation1.pdf]

Results VO2 and HR (supplement)

Repeated measures ANOVAs revealed that there was no significant difference between RCP, m[HHB] $_{BP}$, and EMG$_{BP}$ in heart rate ($F(4,44) = 1.01$, $p = 0.414$, $\eta^2p = 0.084$) and VO_2 ($F(4,44) = 0.53$, $p = 0.71$, $\eta^2p = 0.046$).

The mean average difference between RCP and m[HHB]$_{BP}$ heart rate was 2.68 bpm (limits of agreement (LoA): lower = -11.5, higher = 16.9 bpm) with a mean absolute difference of 5.9 ± 4.7 bpm. The mean average difference between RCP and EMG $_{BP}$ was -0.5 bpm (LoA: lower = -9.2, higher = 8.1 bpm) with a mean absolute difference of 3.4 ± 2.7 bpm. The mean average difference between m[HHB]$_{BP}$ and EMG $_{BP}$ was 3.2 bpm (LoA: lower = -16.9, higher = 23.4 bpm) with a mean absolute difference of 7.7 ± 7.2 bpm. Heart rate values correlated significantly ($p < 0.05$) with each other. The correlation coefficients for heart rates at breakpoints were $r = 0.76$ (m[HHB]$_{BP}$ VL loc vs EMG$_{BP}$ VL loc), $r = 0.88$ (m[HHB]$_{BP}$ VL Loc vs. RCP), and $r = 0.96$ (RCP vs. EMG$_{BP}$ VL Loc). Bias assessed as regression intercepts were not significant ($p = 0.41 – 0.74$).

The mean average difference between RCP and m[HHB]$_{BP}$ VO_2 was 0.10 l/min (limits of agreement (LoA): lower = -0.38, higher = 0.59 l/min) with a mean absolute difference of 0.21 ± 0.16 bpm. The mean average difference between RCP and EMG $_{BP}$ was 0.02 l/min (LoA: lower = -0.45, higher = 0.50 l/min) with a mean absolute difference of 0.19 ± 0.15 l/min. The mean average difference between m[HHB]$_{BP}$ and EMG $_{BP}$ was 0.08 l/min (LoA: lower = 0.55, higher = 0.74 l/min) with a mean absolute difference of 0.24 ± 0.24 l/min. VO_2 values correlated significantly ($p < 0.05$) with each other. The correlation coefficient for VO_2 at breakpoints were $r = 0.67$ (m[HHB]$_{BP}$ VL loc vs EMG$_{BP}$ VL loc), $r = 0.81$ (m[HHB]$_{BP}$ VL Loc vs. RCP), and $r = 0.83$ (RCP vs. EMG$_{BP}$ VL Loc). (see Fig. 5/6 suppl.) Bias assessed as regression intercepts were not significant ($p = 0.17 – 0.21$).

Figure captions (supplement)

Fig. 5 (suppl): Bland-Altman plots displaying agreement between heart rate (upper panel) resp. VO_2 (lower panel) corresponding with RCP and VL loc mHHB (A resp. D), RCP and VL Loc EMG BP (B resp. E), and VL Loc EMG BP and VL Loc mHHB (C resp. F). The horizontal solid line represents the mean difference and the horizontal dashed line the 95% limits of agreement.

Figure 6 (suppl.): Relation of HR (upper panel) resp. VO_2 (lower panel) between m[HHB]$_{BP}$ and RCP (A resp. D), EMG$_{BP}$ and RCP (B resp. E), and m[HHB]$_{BP}$ and EMG$_{BP}$ (C resp. F) in the locomotor VL including Pearson correlation coefficients (all $p<0.05$). The dashed lines represent the line of identity.
