# Supplementary figures and images for: The relationship and agreement between systemic and local breakpoints in locomotor and non-locomotor muscles during single-leg cycling
Source: Front Physiol. 2025 Feb 24;16:1465344. doi: 10.3389/fphys.2025.1465344 (PMC11891192; doi:10.3389/fphys.2025.1465344)

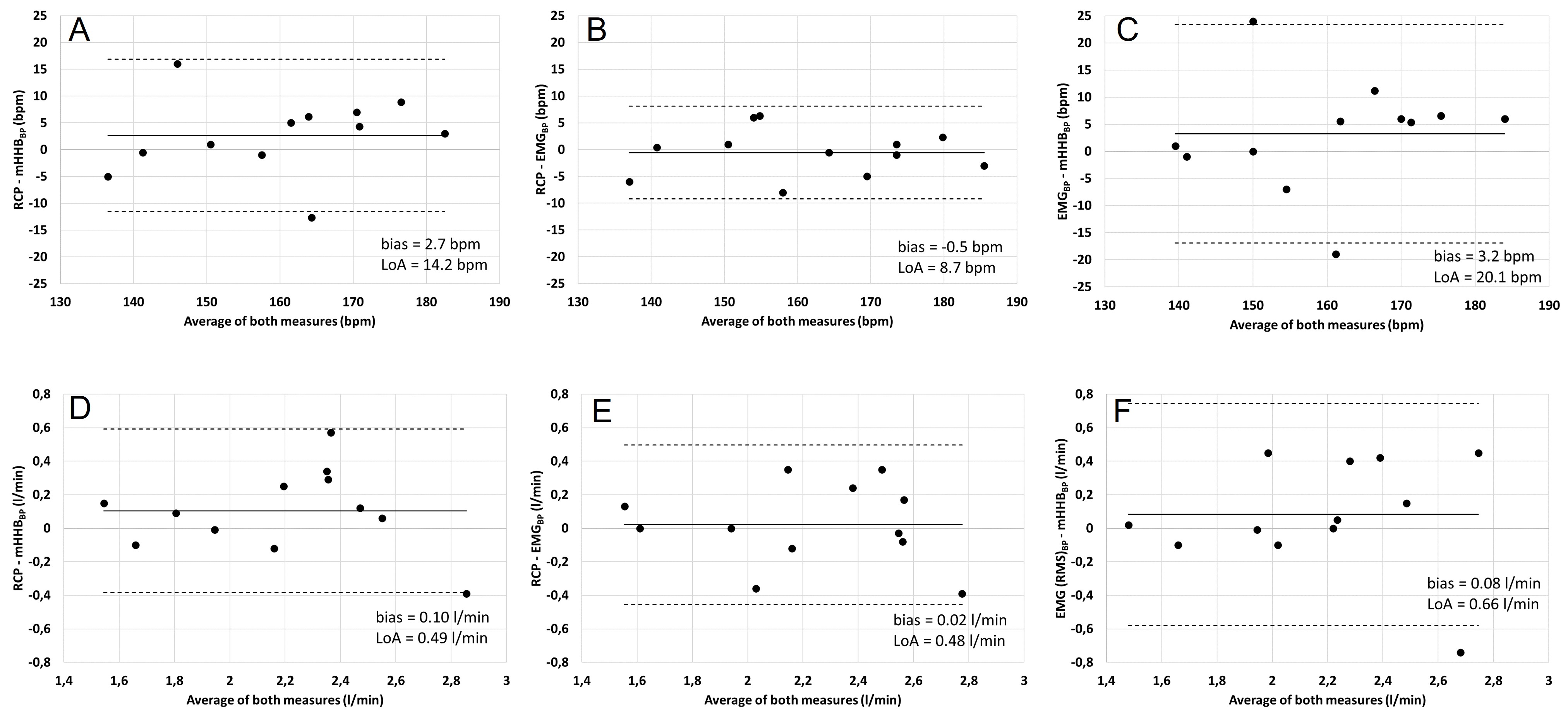

Supplement: Supplementary file 2 [file Image1.jpeg]

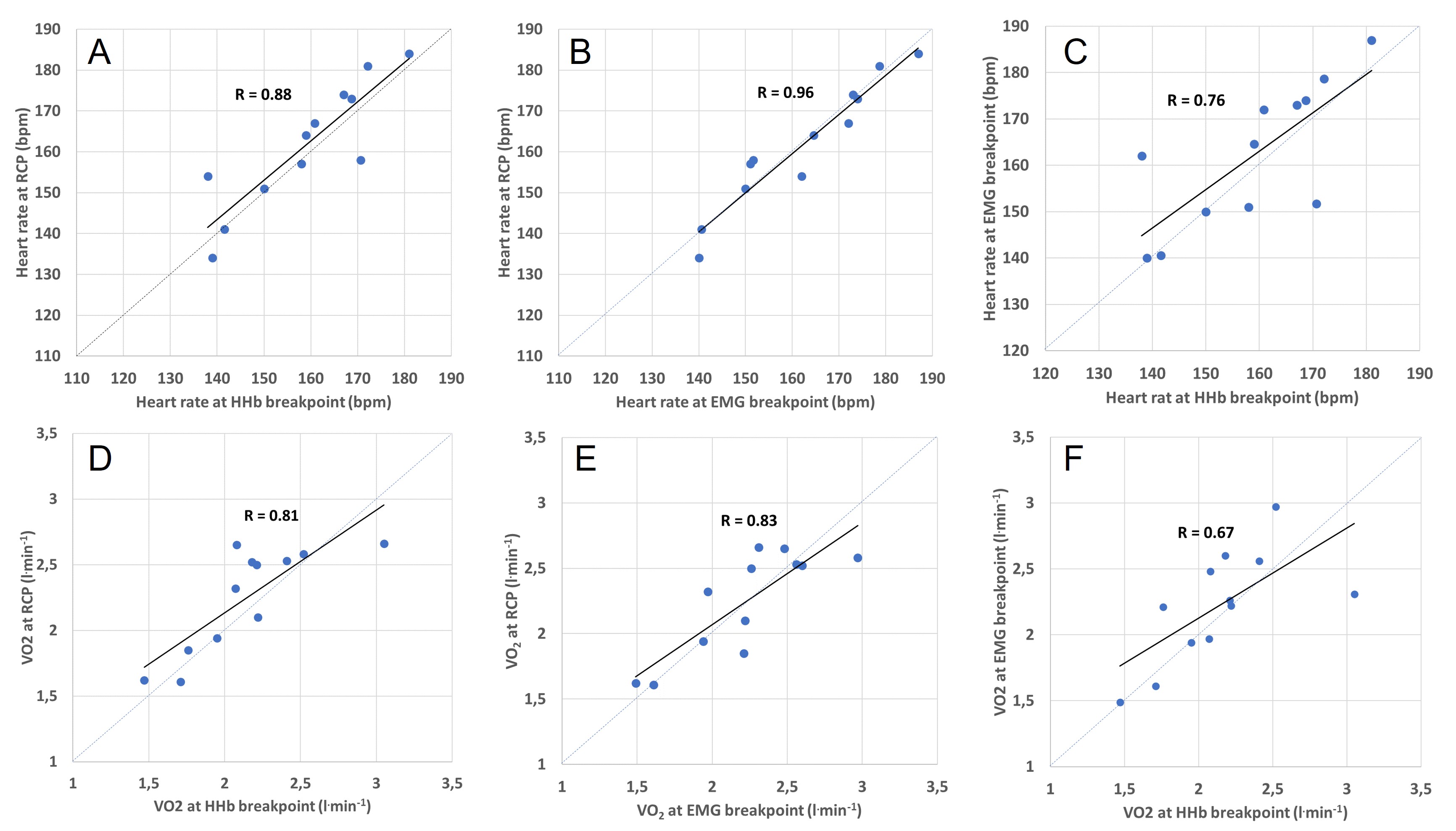

Supplement: Supplementary file 3 [file Image2.jpeg]
